# Texture features from computed tomography correlate with markers of severity in acute alcohol-associated hepatitis

**DOI:** 10.1038/s41598-020-74599-4

**Published:** 2020-10-21

**Authors:** Michele M. Tana, David McCoy, Briton Lee, Roshan Patel, Joseph Lin, Michael A. Ohliger

**Affiliations:** 1 Division of Gastroenterology, Department of Medicine, University of California San Francisco, San Francisco, CA USA; 2 Zuckerberg San Francisco General Hospital and Trauma Center, San Francisco, CA USA; 3 University of California San Francisco Liver Center, San Francisco, USA; 4 Department of Radiology and Biomedical Imaging, University of California San Francisco, San Francisco, CA USA; 5 School of Medicine, University of California San Francisco, San Francisco, CA USA; 6 Department of Surgery, University of California San Francisco, San Francisco, CA USA

**Keywords:** Anatomy, Gastroenterology, Hepatology, Hepatitis, Computer science, Software

## Abstract

The aim of this study was to use texture analysis to establish quantitative CT-based imaging features to predict clinical severity in patients with acute alcohol-associated hepatitis (AAH). A secondary aim was to compare the performance of texture analysis to deep learning. In this study, mathematical texture features were extracted from CT slices of the liver for 34 patients with a diagnosis of AAH and 35 control patients. Recursive feature elimination using random forest (RFE-RF) was used to identify the best combination of features to distinguish AAH from controls. These features were subsequently used as predictors to determine associated clinical values. To compare machine learning with deep learning approaches, a 2D dense convolutional neural network (CNN) was implemented and trained for the classification task of AAH. RFE-RF identified 23 top features used to classify AAH images, and the subsequent model demonstrated an accuracy of 82.4% in the test set. The deep learning CNN demonstrated an accuracy of 70% in the test set. We show that texture features of the liver are unique in AAH and are candidate quantitative biomarkers that can be used in prospective studies to predict the severity and outcomes of patients with AAH.

## Introduction

Acute alcohol-associated hepatitis (AAH) is a major clinical challenge and is characterized by hepatic and systemic inflammation in patients who drink excessive amounts of alcohol^1^. In severe AAH, 1-month mortality rates can be as high as 50%, caused in part by impaired liver regeneration and systemic inflammatory response syndrome (SIRS)^2,3^. The gold standard for diagnosis of AAH is liver biopsy^4,5^, where characteristic findings include presence of neutrophilic lobular inflammation and hepatocyte degeneration as marked by Mallory-Denk bodies^6^. However, because of its associated risks of infection, cost, and lack of availability in some communities, liver biopsy is rarely performed in the clinical care of suspected AAH. Currently, scores such as Maddrey’s Discriminant Function and Model of End Stage Liver Disease (MELD) are used to help clinicians decide if medical therapy is warranted^7,8^. However, because clinical outcomes in severe AAH are poor and alcohol-related deaths are increasing in the United States, there is a great need to develop additional markers and tools to risk-stratify cases of AAH^9,10^. These developments could also contribute to earlier detection of less severe AAH as treatment options are limited and have questionable efficacy for very severe AAH^11,12^. In the few patients who are started on medical therapy for AAH, it is not until day 7 of corticosteroid therapy that treatment response is assessed to determine if the full month-long course should be completed^13^.

Imaging is not part of the standard workup of patients with known AAH, but imaging studies are frequently obtained when patients present to the emergency department with acute symptoms in order to exclude other processes, such as cholecystitis or pancreatitis. Imaging findings in AAH are generally regarded as nonspecific, contributing to neither diagnosis nor management. One study modeled radiologic features with clinical presentation data to predict biopsy-confirmed AAH and found that greater leukocyte count at admission and liver surface nodularity were strong predictors of biopsy-confirmed AAH^14^. With these individual predictors, the logistic model in the validation set showed a specificity of 86% but a sensitivity of only 59%. Furthermore, the image metrics (hepatomegaly, presence of ascites, and portal vein thrombosis) and biomarkers used in that study and other similar studies had limited diagnostic value^15,16^.

In this study, our goal is to determine whether liver texture features derived from CT images could provide improved, noninvasive, surrogate metrics that correlate with clinical data and prognosis in patients with AAH. Texture analysis is a quantitative method of assessing the relationship and distribution of pixel intensities on biomedical images^17^. This method has been applied to the diagnosis of spinal cord myelopathy^18^, the characterization of lung cancers^19–22^, and the identification of liver cirrhosis and malignancy^23–25^ but has not been studied in the context of AAH. Texture analysis utilizes high-dimensional data, for which standard parametric statistics are not suited. Therefore, in the current study, we use machine learning methods (such as random forest and elastic net regression) to develop a texture-based algorithm. The most relevant texture features that distinguished patients with AAH from controls are first determined and then used in a model to quantitatively predict laboratory and other clinical features of the patients. Additionally, we developed a deep learning two-dimensional convolutional neural network (2D CNN) to compare to the texture-based algorithm in distinguishing AAH.

We hypothesize that texture features extracted from CT data will be different in AAH patients compared to controls. We also hypothesize that these quantitative CT texture features will correlate with relevant clinical markers of liver disease severity, potentially providing a novel non-invasive radiomic biomarker to assist in diagnosis, prognosis and management. We also hypothesized that deep learning could be used to accurately and automatically detect AAH, with results comparable to a texture-based algorithm. While our long-term goal is to use quantitative texture features to non-invasively determine the severity of AAH for treatment guidance, this initial proof-of-principle study seeks to first determine whether texture features were related to clinically measurable parameters. This study was approved by the University of California San Francisco Institutional Review Board (IRB, 14-13492). All methods were carried out in accordance with relevant guidelines and regulations.

## Results

### Participant characteristics

Thirty-four AAH patients met the inclusion criteria over the 38-month study period. Twenty-nine AAH patients (85.3%) were men, mean age at diagnosis was 43.4 years, and mean Maddrey’s Discriminant Function score was 38. Twenty-two patients eventually received treatment for AAH, including receiving corticosteroids only (n = 8), pentoxifylline only (n = 10), and a combination of corticosteroids and pentoxifylline (n = 4). The patients’ characteristics are summarized in Table 1. Thirty-five trauma patients served as controls. Twenty-five control patients (66.7%) were men, and mean age at presentation was 44.2 years. As measures of outcome, day 7 bilirubin was available for 28 AAH patients, and day 30 bilirubin was available for 19 patients. Among those patients with available data, day 7 bilirubin was on average 0.3 mg/dL higher than baseline (SD 4.4 mg/dL). Day 30 bilirubin was on average 2.3 mg/dL lower than baseline (SD 9.1 mg/dL). Among patients who received treatment, the mean change in total bilirubin to day 7 was + 0.2 mg/dL; for patients who did not receive treatment, the mean change in total bilirubin to day 7 was + 0.5 mg/dL. Among patients who received treatment, the mean change in total bilirubin to day 30 was − 3.2 mg/dL; for patients who did not receive treatment, the mean change in total bilirubin to day 30 was + 1.1 mg/dL. Neither the change in bilirubin at day 7 nor at day 30 differed significantly between AAH patients who received treatment and did not receive treatment (p = 0.80, p = 0.40). Patients with AAH were followed for a median of 28.7 months, and 5 patients died during follow-up.

**Table 1:** Demographic and laboratory characteristics of trauma control and acute alcohol-associated hepatitis (AAH) patients.

	Control	AAH	
	n = 35	n = 34	
**Gender (%)**			p < 0.01
Female	10 (33.3)	5 (14.7)	
Male	25 (66.7)	29 (85.3)	
**Mean age (years)**	44.2	43.4	p = 0.75
**Race/ethnicity (%)**			p = 0.22
White	9 (25.7)	21 (26.5)	
Black or African American	5 (14.3)	6 (8.8)	
Latino or Hispanic	15 (42.9)	32 (58.8)	
Asian Pacific Islander	4 (11.4)	–	
Other/unknown	2 (5.7)	2 (5.9)	
**Median lab values at presentation**			p < 0.01
AST (U/L)	32.5	153	
ALT (U/L)	28	48	
Total Bilirubin (mg/dL)	0.5	12.0	
INR	1.0	1.7	

*AST* aspartate aminotransferase, *ALT* alanine aminotransferase, *INR* international normalized ratio.

### Results of RFE-RF

**Figure 1:**
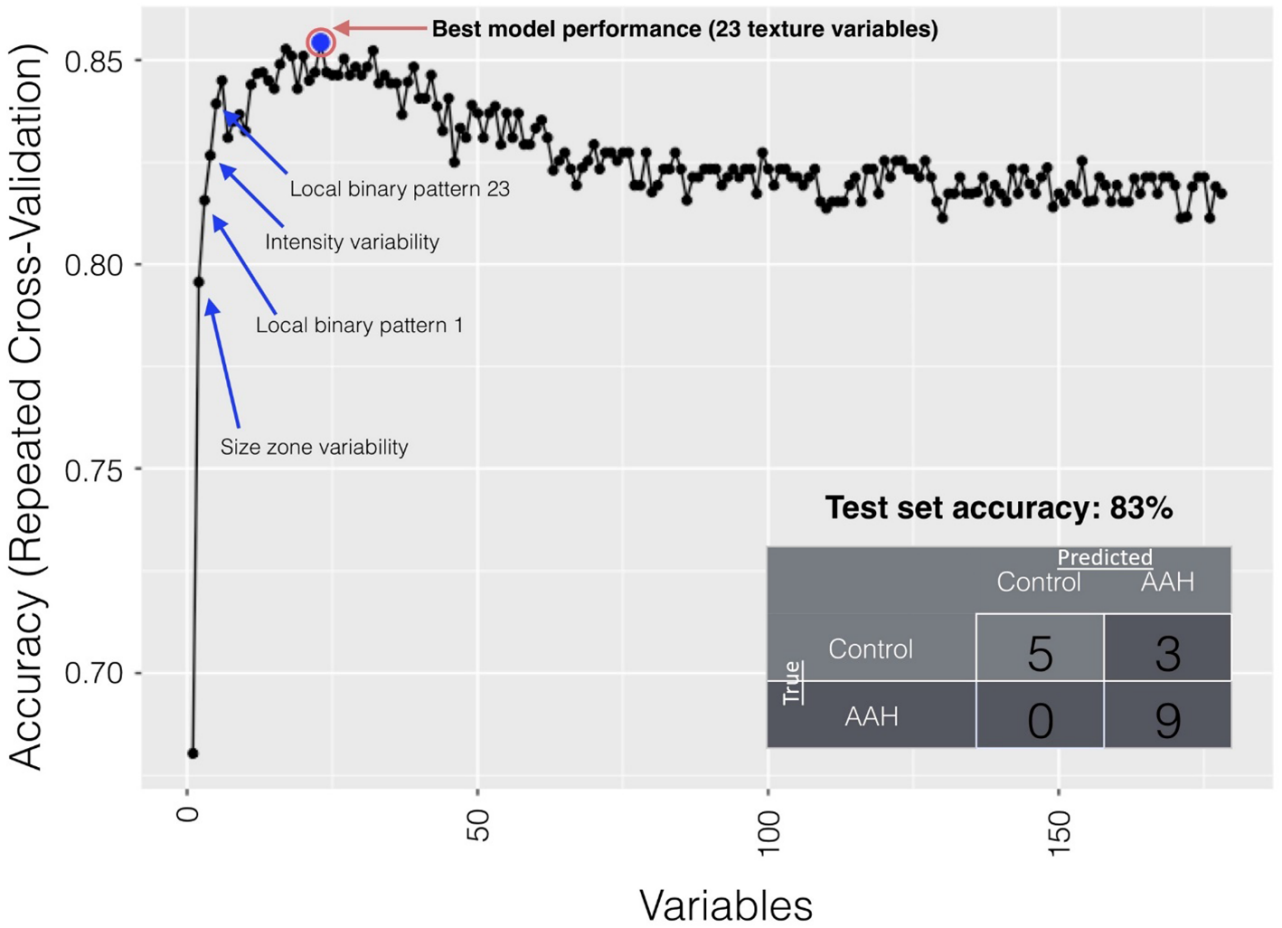
Results from the Recursive Feature Elimination using Random Forest (RFE-RF) algorithm, used to identify key CT liver texture features to differentiate between AAH and control patients. A total of 178 texture features, and a combination of 23 of these features were associated with the best model performance (85.4% accuracy in distinguishing AAH from control patients). These 23 texture features are listed in Table 3. The 23-feature model applied to the left-out test set is described in the bottom-right table and resulted in an accuracy of 82.4% (14 of 17 patients), sensitivity of 100%, specificity of 75%, NPV of 100%, and PPV of 62.75%.

Of the 178 liver features extracted by texture analysis, the recursive feature elimination using random forest (RFE-RF) classification found that the model performed best in distinguishing AAH cases from controls with 23 specific features. Table 2 describes the top texture features. The combination of these features in a texture-based algorithm yielded an accuracy of 85.4% in distinguishing AAH cases from controls in the training data (Fig. 1). The top texture features were gray level size zone variability, gray level non-uniformity (GLNU), run length non-uniformity (RLN), mean deviation, kurtosis, cluster tendency, short run emphasis, mean deviation, inverse variance and eleven metrics from the local binary pattern matrix (LBM) (Table 3). Applying the 23-feature model to make AAH predictions using data from the left-out test set resulted in an accuracy of 82.4% (14 of 17 patients), sensitivity of 100%, specificity of 75%, NPV of 100%, and PPV of 62.75%.

**Table 2:** Definition of top texture features.

Feature name	Definition
Mean deviation	Mean of the absolute deviation of the pixel intensity around the mean
Local binary pattern matrix (LBM)	Bins of binary pattern distributions around a pixel from local binary pattern of matrix calculations
Run length non-uniformity (RLN)	Measures the similarity of gray level runs in a determined degree direction. A gray level run is a set of consecutive, collinear pixels having the same gray level. The RLN is low if the run lengths are alike
Gray level non-uniformity (GLNU)	Total non-uniformity of pixel intensities throughout the region of interest. It measures the similarity of gray level intensity values in the image. The GLNU is low if the intensity values are alike
Short run emphasis	This metric increases when gray level short runs are predominant
Kurtosis	Measure of peakedness of pixel distribution
Size zone variability	Variability found in gray level size zone matrix generated from image
Cluster tendency	Measure of tendency of data to form non-random clusters
Inverse variance	Inverse of the measure of distribution around the mean

**Table 3:** Results of elastic-net regression and linear modeling of the top texture features and their most closely associated clinical predictor.

Texture feature	Elastic net RMSE	Top predictor	Linear model coefficient	Linear model P-value	FDR P-value correction
0 GLNU	1.96 × 10^3^	WBC	6.74 × 10^1^	0.047	0.083
**90 GLNU**	**1.92 × 10**^**3**^	**WBC**	**8.15 × 10**^**1**^	**0.022**	**0.049**
**0 RLN**	**2.63 × 10**^**3**^	**AST**	**− 1.16 × 10**^**1**^	**0.009**	**0.035**
**45 RLN**	**3.75 × 10**^**3**^	**AST**	**− 1.65 × 10**^**1**^	**0.006**	**0.029**
**90 RLN**	**3.25 × 10**^**3**^	**AST**	**− 1.45 × 10**^**1**^	**0.006**	**0.029**
**135 RLN**	**3.82 × 10**^**3**^	**AST**	**− 1.49 × 10**^**1**^	**0.019**	**0.049**
**135 cluster tendency**	**6.76 × 10**^**–1**^	**AST**	**− 3.02 × 10**^**–3**^	**0.003**	**0.020**
**Kurtosis**	**5.47**	**AST**	**2.71 × 10**^**–2**^	**0.001**	**0.011**
LBM 0	4.40 × 10^–3^	WBC	1.28 × 10^–4^	0.095	0.126
LBM 1	2.10 × 10^–3^	AST	5.60 × 10^–6^	0.083	0.120
LBM 2	1.50 × 10^–3^	WBC	3.67 × 10^–5^	0.147	0.161
**LBM 13**	**2.00 × 10**^**–4**^	**Albumin**	**− 1.14 × 10**^**–4**^	**0.012**	**0.040**
LBM 18	1.00 × 10^–4^	Platelet count	− 4.62 × 10^–6^	0.226	0.226
LBM 20	3.00 × 10^–4^	Platelet count	− 1.48 × 10^–5^	0.102	0.126
LBM 21	6.00 × 10^–4^	WBC	3.52 × 10^–5^	0.110	0.126
LBM 22	1.30 × 10^–3^	WBC	1.28 × 10^–5^	0.215	0.225
LBM 23	2.10 × 10^–3^	AST	6.13 × 10^–6^	0.047	0.083
LBM 24	4.83 × 10^–2^	WBC	− 1.24 × 10^–3^	0.105	0.126
LBM 25	3.14 × 10^–2^	AST	8.04 × 10^–5^	0.072	0.111
Mean deviation	3.09 × 10^–2^	WBC	9.81 × 10^–4^	0.072	0.111
**45 short run emphasis**	**6.94 × 10**^**–2**^	**AST**	**− 4.28 × 10**^**1**^	** < 0.001**	**0.049**
Size zone variability	3.95 × 10^2^	Blood urea nitrogen	2.11 × 10^1^	0.034	0.071
**Inverse variance**	**5.87 × 10**^**2**^	**Cirrhosis**	**2.58 × 10**^**2**^	**0.021**	**0.049**

Texture features with corrected p-values meeting statistical significance are bolded.

*RMSE* root mean square error, *FDR* false discovery rate, *GLNU* gray level non-uniformity, *RLN* run length non-uniformity, *LBM* local binary pattern matrix, *AST* aspartate aminotransferase, *ALT* alanine aminotransferase, *WBC* white blood cell count.

### Results of elastic-net regression

**Figure 2:**
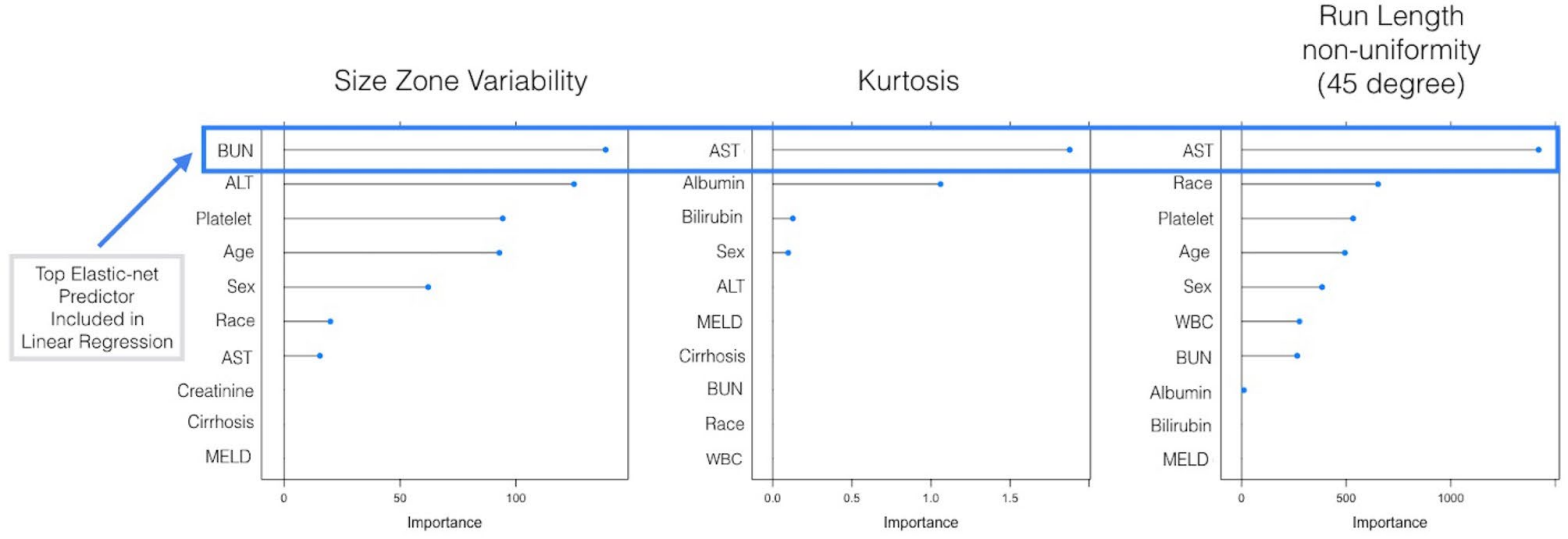
Examples of elastic-net regression outputs for three texture features. For each texture feature, 13 clinical variables were used as predictors. The top elastic-net clinical predictor for each texture feature was identified for testing in a simple linear regression model.

The elastic net regressions using 13 clinical variables as predictors and each of the 23 texture features yielded a single best clinical variable associated with each CT texture feature. Figure 2 shows an example of three elastic models run with liver texture outcomes and top clinical predictors. This association between top clinical features and the texture features was tested in a simple linear regression model (Table 3). The elastic net determined that the best clinical predictors for the top 23 CT texture features distinguishing AAH from controls were white blood cell count (WBC) (best clinical predictor for 8 of the top 23 texture features), aspartate aminotransferase (AST) (10 of 23), cirrhosis (1 of 23), albumin (1 of 23), platelet count (2 of 23), and blood urea nitrogen (1 of 23).

Seven of the top RFE texture features to detect AAH were significantly associated with AST (significant after FDR correction). Measures of liver homogeneity, specifically the RLN (in 0, 45, 90, and 135 degree directions), kurtosis, cluster tendency and short run emphasis were most closely associated with AST. All RLN texture features were negatively associated with AST; on average a one-unit increase AST was associated with a 14.4 reduction of RLN metrics.

### Results of deep learning

**Figure 3:**
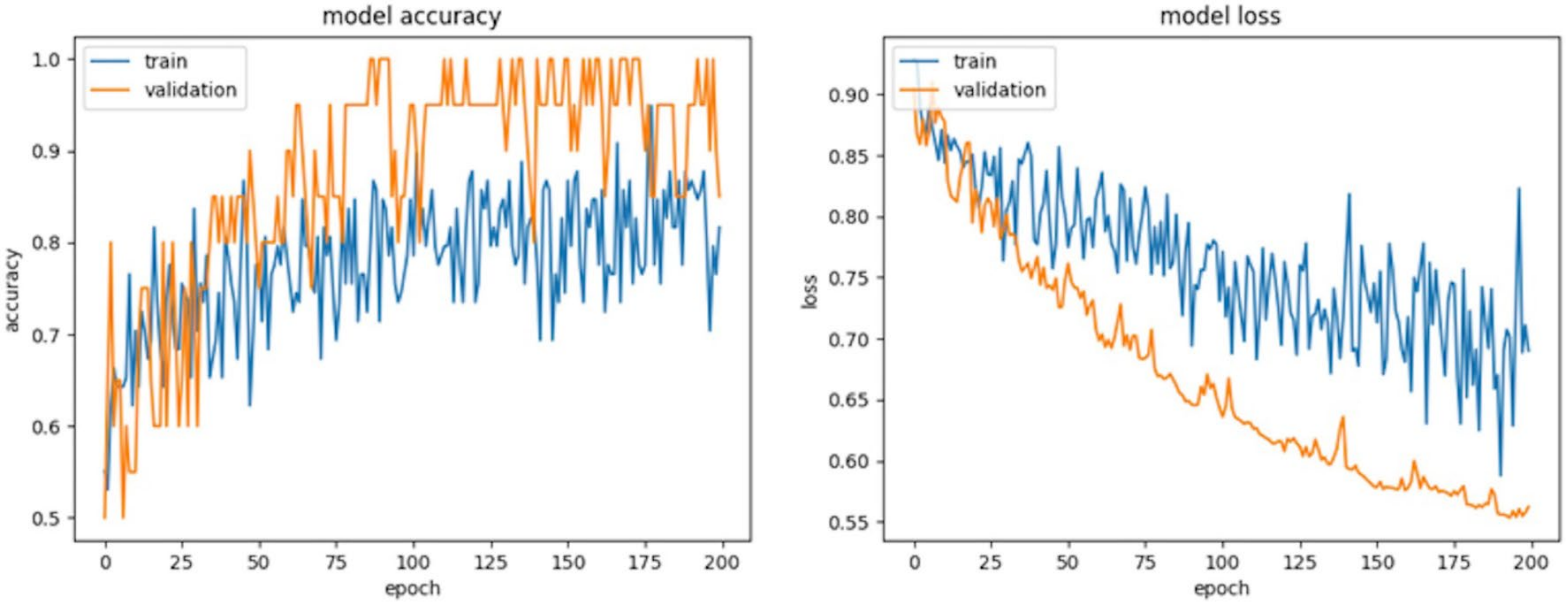
Results for deep learning accuracy and loss over 200 epochs for both the training and validation datasets. The model accuracy in the training set reached 95% accuracy, with overall accuracy of 70%. The validation set reached 100% accuracy. Loss improved as expected over 200 epochs, with the lowest loss of the model’s performance on the test set of 0.56.

Results for deep learning CNN accuracy and loss over the 200 epochs are shown in Fig. 3. The best model based on validation accuracy was used to make predictions from the unused test set data (10 patients). The CNN model’s performance on the test-set was an overall accuracy of 70% with a loss of 0.56; the validation set reached 100% accuracy (on all 10 patients) and the training set reached 95%. Additional measures of the model’s performance on the test set were precision (0.75), recall (0.60), and F1 score (0.66).

## Discussion

In this retrospective single-center study, we have identified CT-based image texture features that distinguish patients with AAH from controls. We also demonstrated that those texture features are quantitatively associated with important clinical parameters, most notably AST. Many of the top texture features showed significant association with the AST, even after correcting for multiple comparisons. Importantly, run length measures of non-uniformity were negatively associated with AST. In particular, increases in AST were associated with overall reductions in run length measures of non-uniformity, suggesting that these CT texture features may reflect the severity of inflammation classically associated with AAH. As machine learning gains momentum in the field of radiology, our study represents a unique contribution showing the correlation of clinical data to liver texture features.

We attempted to distinguish AAH from control images using two methods: (1) machine learning to determine salient texture features, and (2) deep learning to automatically detect AAH. While both methods were comparable, surprisingly our primary texture-based algorithm was found to be more effective. Not only did the 2D CNN underperform in distinguishing AAH from controls when compared to the texture-based algorithm, but like any deep learning method it was also unable to allow for feature extraction. However, our study provides proof that deep learning can identify AAH and offers a foundation to train and validate future deep learning algorithms. Further studies would build upon this CNN, and its accuracy should improve when trained with a larger set of images.

Our study is limited by its small sample size, influencing the training of the algorithms and overall accuracy. Additionally, the diagnosis of AAH was made based on clinical assessment rather than biopsy, as biopsy is rarely performed in the acute setting despite being the gold standard. Our study may also be limited by the fact that some patients received treatment at variable times relative to time of imaging; however, given the limited or uncertain benefit of treatment in AAH, imaging is likely to be unaffected in the short-term. We used laboratory values that were contemporaneous with the CT scans, so the correlation of laboratory values with the CT should relate to the clinical state at the time the image was acquired. Additionally, if treatment were beneficial, we would expect there to be fewer differences in imaging features. Using an imaging study to predict a common imaging test is, by itself, limited. Further studies will be required to determine whether these texture features are able to prospectively predict the amount of liver injury in patients and/or predict clinical outcomes such as short-term mortality or response to treatment. It should be noted that trauma patients represent an imperfect control group because trauma can be associated with alcohol use. However, we would expect any occult alcohol-associated injury in the control group to lead us to underestimate the significance of our results.

The current clinical standard for diagnosis of acute hepatitis by imaging is qualitative assessment by a radiologist. Indeed, acute hepatitis does have many imaging characteristic features that may be detected on CT examinations. However, our goal in examining texture features was to generate a quantitative tool that could eventually be used to quantitatively grade severity and potentially follow patients over time.

The machine learning pipeline used in this work was entirely based on non-proprietary, publicly available software, which increases the potential applicability of the method. Furthermore, the unbiased region of interest analyzes an entire axial 2D slice which lends itself to automated analysis. Future directions include identification of liver texture features associated with survival rates (which could be assessed with Poisson or Cox regression), comparison of additional control groups, distinguishing AAH from decompensated alcohol-associated cirrhosis, and evaluating three-dimensional (3D) CNN to make use of features in the z-dimension.

In summary, we have shown that CT texture features correlate with clinical parameters in AAH, suggesting that the application of this novel tool may assist in the diagnosis of AAH and guide treatment. This was a proof-of-principle study, without sufficient follow-up data to correlate texture features with clinical outcomes. This pilot study demonstrates that CT texture analysis has promise as a prognostic tool in AAH and could potentially guide management by identifying clinical phenotypes.

## Methods

### Study population

**Figure 4:**
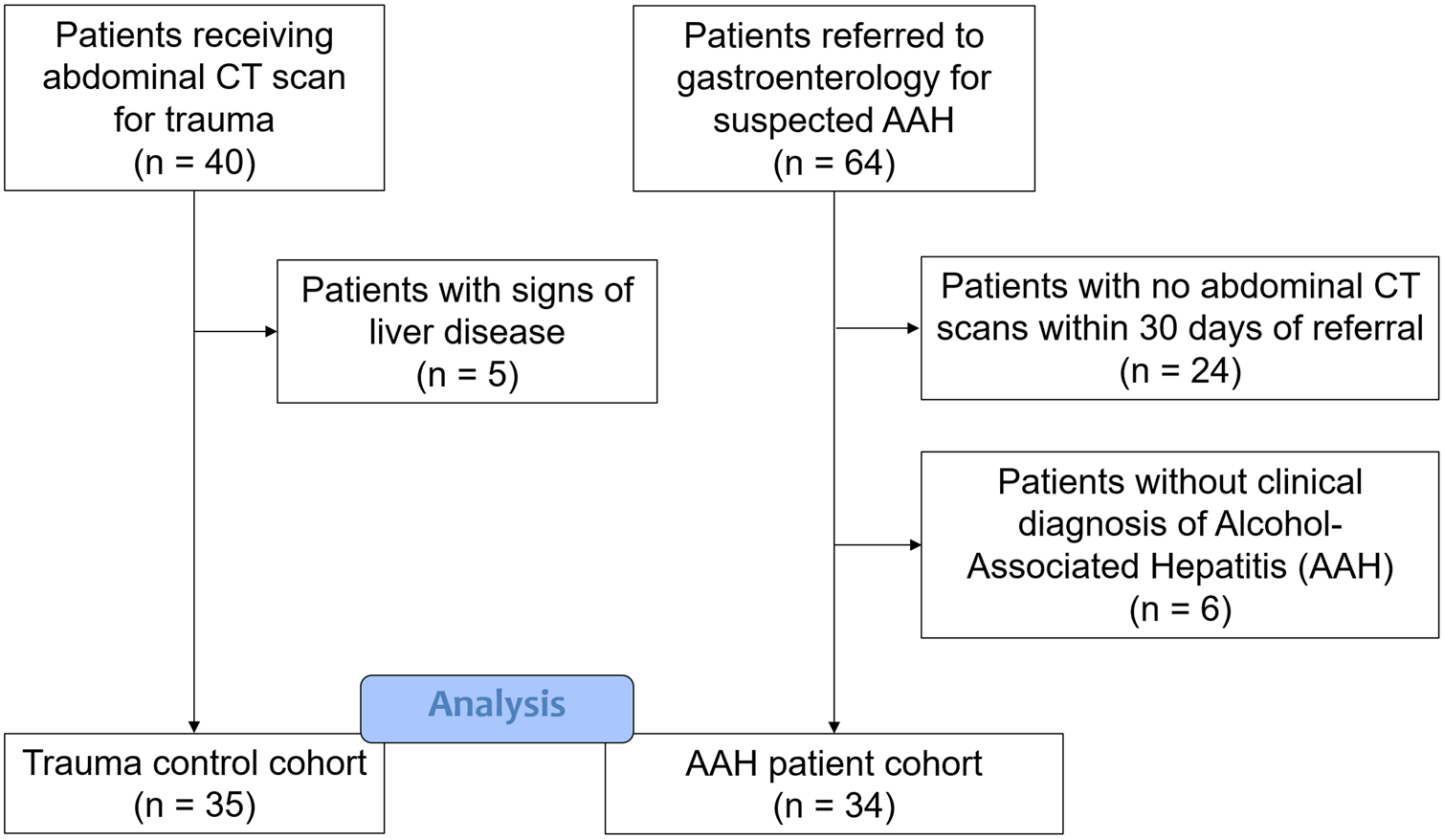
Flowchart demonstrating the inclusion and exclusion criteria used to create our cohorts of AAH and control patients.

This was a single-center retrospective study based on review of medical records. The study population included patients admitted to an urban safety-net county hospital from September 2013 through November 2016 who had received a diagnosis of AAH. At our institution, AAH is a diagnosis typically made based on clinical impression. Supporting data include a history of excessive alcohol consumption; clinical presentation (including abdominal pain, malaise); physical exam findings of fever, jaundice, tender hepatomegaly, ascites, and hepatic encephalopathy; laboratory values such as leukocytosis and thrombocytosis, hyperbilirubinemia, and AST:ALT elevation in a ratio ≥ 2:1. Patients were included in the study if they were referred to the inpatient gastroenterology consultation service, received a clinical diagnosis of AAH, and underwent a contrast CT scan of the abdomen within a month of that diagnosis. For imaging controls, we considered those who had received a CT scan for suspected trauma but without abdominal injury. Trauma patients with signs of liver disease, such as fatty liver or cirrhosis were excluded from the control group. Figure 4 is a flowchart that demonstrates the selection of our cohorts by inclusion and exclusion criteria. Demographic information, clinical presentation, laboratory data, treatment information, and outcomes were reviewed for each patient. Pertinent laboratory values were chosen as close as possible from the time of the CT. This study was compliant with the health insurance portability and accountability act (HIPAA) and was approved by the local institutional review board for human research. Informed consent was waived. All clinical data were stored in a secure research electronic data capture program^26^.

### CT acquisition parameters

All CT images were acquired using a standard clinical protocol on a 64-slice CT scanner (Lightspeed, GE Healthcare) following administration of approximately 150 mL of iodinated contrast (Omnipaque-350) with a tube potential (kVp) of 120 keV and automatic tube current modulation. Contiguous axial images with a slice thickness of 1.25 mm were obtained in the portal venous phase of intravenous contrast enhancement and were reconstructed using filter back projection and a soft-tissue reconstruction kernel.

### Image annotation

For each of the 34 AAH patients and 35 control subjects, a single axial CT slice at the level of the right portal vein bifurcation was chosen for analysis. The image was manually segmented using imageJ software to include the entire axial liver slice^27^. The inferior vena cava and hilar vessels were excluded. Initial liver segmentation was performed by a radiology data scientist and validated by a fellowship trained abdominal radiologist. CT imaging data were divided into 52 training images and 17 testing images for machine learning analysis. For 2D CNN analysis, image data were randomly split into training (49), validation (10), and testing (10) sets.

### Texture analysis

**Figure 5:**
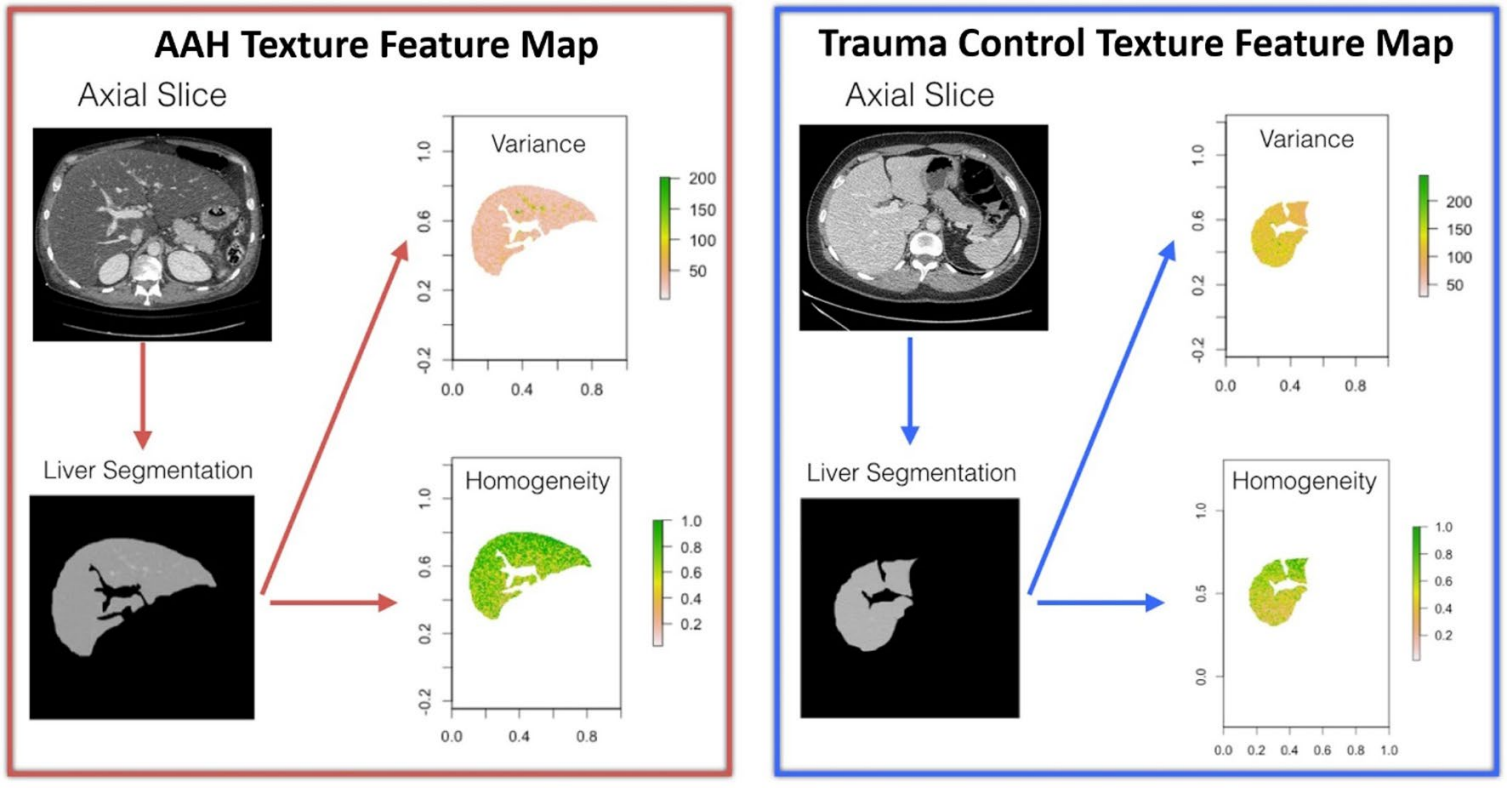
Example of the texture analysis pipeline, which is identical for both AAH (left) and control (right) patients. The chosen CT axial slice was selected to be at the level of the right portal vein bifurcation for each patient. The liver was manually segmented from the axial slice. Texture features were then extracted from liver segments. Maps of representative texture features (variance and homogeneity) are shown.

Grayscale values for the liver axial 2D image were analyzed using custom software written in Python and R. The scikit-image package^28^ in Python was used to calculate the local binary patterns imbedded in liver texture. These results and images were transferred and processed in R using the radiomics package^29,30^. Calculations were made for the (i) First-Order Statistics, (ii) Gray Level Co-occurrence Matrix, (iii) Gray Level Run Length Matrix, and (iv) Gray Level Size Zone Matrix for each direction (0, 45, 90, and 135 degrees). Before analysis, all images were discretized to values between zero and eight. This yielded 178 texture features from each image, with each feature summarized as a single digit. The overall texture analysis pipeline is illustrated in Fig. 5.

### Machine learning

Recursive feature elimination using RFE-RF from the Classification and Regression Training (caret) package^31^ in R was first used to identify the key CT liver texture features that delineate AAH from controls. We performed tenfold cross validation, which was repeated 10 times, during the training to provide robust and stable estimates^32^. Additionally, 20% of the data were left out of training and used as a left-out test set. Texture variables identified as the top features from the RFE-RF were used in subsequent analysis to identify the association between these liver texture features and clinical presentation data. The performance of the classifier in this stage of analysis was evaluated by overall accuracy, sensitivity, specificity, negative predictive value (NPV), and positive predictive value (PPV) from test set predictions.

In order to determine the association between the liver features identified by RFE-RF and clinical laboratory and demographic data, each of the most identified and important texture features was used as an outcome in a series of elastic net regressions. We used 13 laboratory and demographic parameters as predictors: age at diagnosis, race, sex, WBC, blood urea nitrogen, creatinine, total bilirubin, albumin, aspartate aminotransferase (AST), alanine aminotransferase (ALT), platelet count, MELD score, and cirrhosis. Elastic-net regression was used due to the high dimensionality of the predictor variables compared to the number of patients and the multicollinearity of the laboratory values. Furthermore, because elastic-net regression utilized both L1 and L2 penalization, coefficients could be shrunk to zero, which allowed for key clinical variable selection that reduced the mean-squared-error (MSE) in the predicted texture feature. Finally, standard linear regression models were used to determine the magnitude and significance of the association between key clinical parameters and the top liver texture features. There have been a number of statistical studies aimed at determining the correct techniques for ascertaining p-values and unbiased estimates from models utilizing L1 and/or L2 norms^33,34^. Because we were interested in attaining p-values and association estimates without bias, and due to the low sample size, we chose a standard ‘debiasing’ approach after variable selection with lasso regression. The top predictor was included in a simple univariate linear regression model to determine the individual association the clinical indicator had with the texture feature. We applied the false discovery rate (FDR) correction for multiple comparisons.

### Deep learning model

**Figure 6:**
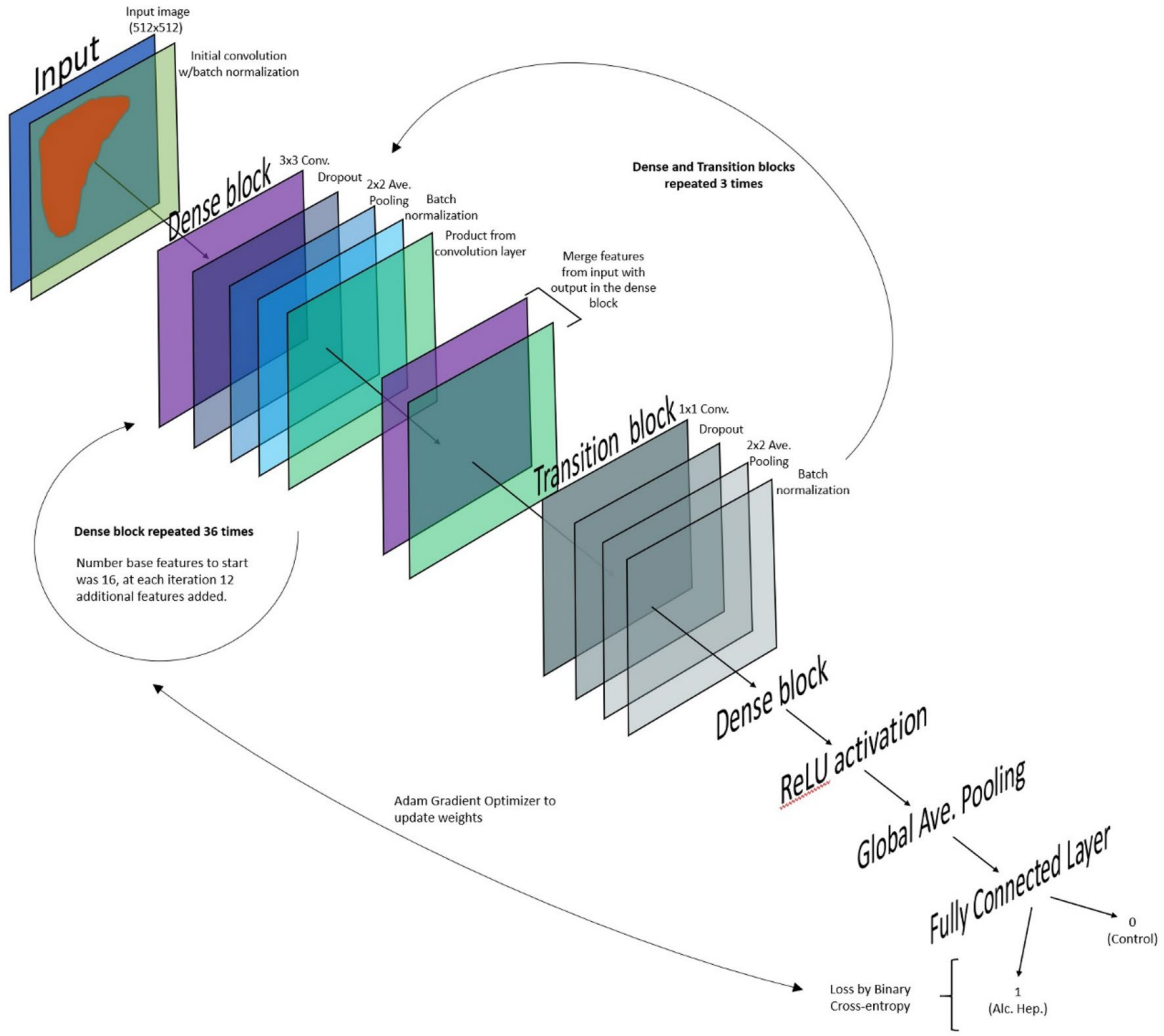
Illustration of the DenseNet architecture used to create a 2D convolutional neural network (CNN) to automatically detect acute AAH. The image of the liver segment undergoes successive layers of 2D convolution, batch normalization, rectified linear unit (ReLU) activation and 2D max pooling. The feature maps for each layer were concatenated with those of the previous layer. The last layer was fully connected with a ReLU activation function, followed by random dropout and prediction.

A 2D CNN using the DenseNet architecture^35^ was created to test the ability of a deep learning algorithm to automatically detect AAH without feature engineering. The model architecture is illustrated in Fig. 6 and included successive layers of (i) 2D convolution, (ii) batch normalization^36^, (iii) rectified linear unit (ReLU) activation function^37^ and (iv) 2D max pooling. The feature maps for each layer *t* were concatenated with those of layer *t* − 1. The last layer was fully connected with a ReLU activation function, followed by random dropout^38^ and prediction. Training was performed with an Adam Optimizer^39^. All computations (parameters optimization, training, and testing) were performed on two NVIDIA 1080ti GPUs and implemented in Python 2.7 using Tensorflow and Keras frameworks. No additional hyper-parameter optimizations were conducted. Measures of the model’s performance on the test set were evaluated by overall accuracy, precision, recall, and F1 score.

### Deep learning data augmentation

Due to the limited image data (34 AAH images and 35 control images) data augmentation was used to avoid overfitting using the Keras framework. Images were augmented using random rotation (20 degrees), width and height shift (10% of image dimensions), random shearing (intensity of 0.2), random zoom (20% of image dimensions), and random vertical/horizontal flipping. Additionally, dropout in the fully connected layer also provides a form of data augmentation.
